# Regulation of the Na,K-ATPase Gamma-Subunit FXYD2 by Runx1 and Ret Signaling in Normal and Injured Non-Peptidergic Nociceptive Sensory Neurons

**DOI:** 10.1371/journal.pone.0029852

**Published:** 2012-01-13

**Authors:** Stéphanie Ventéo, Steeve Bourane, Ilana Méchaly, Chamroeun Sar, Omar Abdel Samad, Sylvie Puech, Rhoda Blostein, Jean Valmier, Alexandre Pattyn, Patrick Carroll

**Affiliations:** 1 INSERM U1051, Institut des Neurosciences de Montpellier, Montpellier, France; 2 Université Montpellier II, Place Eugène Bataillon, Montpellier, France; 3 Université Montpellier 1, Montpellier, France; 4 Departments of Medicine & Biochemistry, Montreal General Hospital Research Institute Montreal, Quebec, Canada; 5 Dana-Farber Cancer Institute, Department of Neurobiology, Harvard Medical School, Boston, Massachusetts, United States of America; University of Cincinnatti, United States of America

## Abstract

Dorsal root ganglia (DRGs) contain the cell bodies of sensory neurons which relay nociceptive, thermoceptive, mechanoceptive and proprioceptive information from peripheral tissues toward the central nervous system. These neurons establish constant communication with their targets which insures correct maturation and functioning of the somato-sensory nervous system. Interfering with this two-way communication leads to cellular, electrophysiological and molecular modifications that can eventually cause neuropathic conditions. In this study we reveal that *FXYD2*, which encodes the gamma-subunit of the Na,K-ATPase reported so far to be mainly expressed in the kidney, is induced in the mouse DRGs at postnatal stages where it is restricted specifically to the *TrkB*-expressing mechanoceptive and Ret-positive/IB4-binding non-peptidergic nociceptive neurons. In non-peptidergic nociceptors, we show that the transcription factor Runx1 controls *FXYD2* expression during the maturation of the somato-sensory system, partly through regulation of the tyrosine kinase receptor Ret. Moreover, Ret signaling maintains *FXYD2* expression in adults as demonstrated by the axotomy-induced down-regulation of the gene that can be reverted by in vivo delivery of GDNF family ligands. Altogether, these results establish FXYD2 as a specific marker of defined sensory neuron subtypes and a new target of the Ret signaling pathway during normal maturation of the non-peptidergic nociceptive neurons and after sciatic nerve injury.

## Introduction

Sensory modalities such as pain, touch and proprioception are relayed from the periphery to the spinal cord by somatosensory neurons located in the dorsal root ganglia (DRGs). These neurons constitute a heterogeneous neuronal population based on anatomical, functional, neurotrophin dependence and molecular criteria. Their cell somas maintain constant communication with their targets via anterograde and retrograde signals that contribute to the maturation and functioning of the system. Damage to the peripheral nerves interferes with this two-way communication, resulting in stereotypic changes in the physiology of DRG neurons, including altered electrical activity, protein activity and gene expression. Identifying genes whose expression is regulated by signaling pathways activated by periphery-derived cues represents one key step toward a better understanding of the establishment, functioning and pathologies of the somatosensory nervous system. In a gene profiling analysis on mouse lumbar DRGs [1], we identified the transcript encoding FXYD2, also known as the gamma-subunit of the NaK-ATPase. The FXYD family comprises 7 members, the main proposed function of which is to modulate the activity of the Na,K-ATPase in different tissues [2], [3]. So far, the major site of *FXYD2* expression has been described in kidney, and human mutations of the *FXYD2* gene lead to renal hypomagnesemia [4]. Here we report the first description of *FXYD2* expression in discrete neuronal sub-populations of the adult mouse DRGs, consisting in the TrkB-positive (+) mechanoceptors and the Ret+/IB4+ non-peptidergic nociceptors. In the latter population, *Runx1* and *Ret* are required for the proper expression of *FXYD2*. Moreover, we establish that *FXYD2* is down-regulated in axotomized neurons, and that in vivo application of the Ret ligands, GDNF or Neurturin prevents this down-regulation in axotomized IB4+ nociceptive neurons. Altogether our results reveal *FXYD2* as a new target of the Ret signaling pathway during normal maturation of the DRG and after sciatic nerve injury in the non-peptidergic nociceptive neurons.

## Results

### *FXYD2* expression is induced in the DRGs at postnatal stages and maintained in the adults

**Figure 1:**
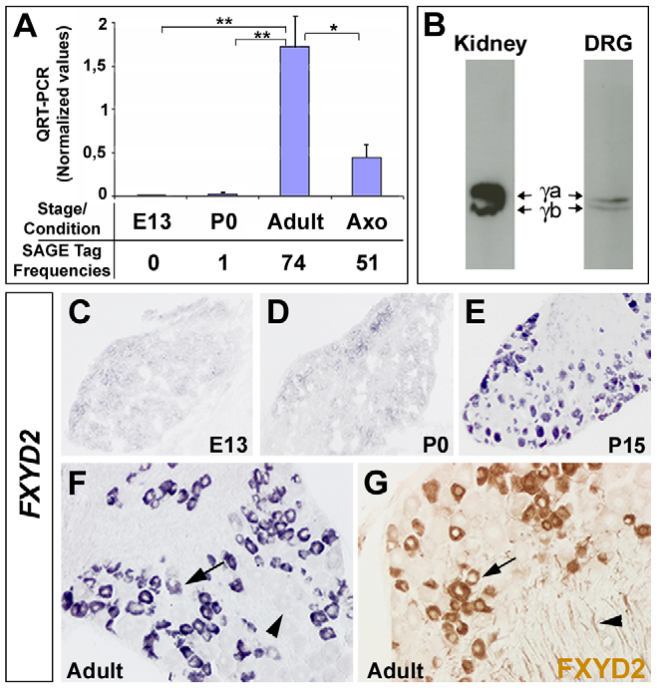
Expression profile of *FXYD2* mRNA and protein during DRG neuron development. (A) Quantitative RT-PCR analysis of *FXYD2* expression in the developing DRGs and after axotomy. SAGE tag frequencies for FXYD2 at equivalent stages or conditions are indicated below. (B) Western blot using a FXYD2 antibody shows the presence of the FXYD2 isoforms gamma-a and gamma-b in the adult DRG. Kidney extract is a positive control. (C–F) *FXYD2* in situ hybridization on mouse DRG sections at E13, P0, P15 and adult. Arrow and arrowhead in F point to FXYD2-positive and FXYD2-negative neurons, respectively. (G) FXYD2 immunochemistry on adult DRG sections. Arrows and Arrowheads point respectively to positive cell bodies and nerve fibers.

Our previous SAGE analysis of DRG transcriptional dynamics [1] allowed the identification of the *FXYD2* gene whose expression appeared virtually absent in the prenatal stages and at birth, peaked in the adult and decreased after axotomy (Fig. 1A). Real-time PCR quantification paralleled the changes in tag frequencies observed by SAGE (Fig. 1A). So far, FXYD2 expression was well characterized in kidneys where 2 isoforms generated by alternative splicing (FXYD2a and FXYD2b) have been described [5]. Western blot analysis on mouse adult DRGs revealed two bands identical to those observed in kidney protein extracts (Fig. 1B), indicating that the two FXYD2 isoforms are also present in the DRGs. To further confirm and extend the transcript quantification results, in situ hybridization was carried out on DRG sections from embryonic day 13 (E13) through adult stages (Fig. 1C–F). As expected, no specific signal was observed at E13 (Fig. 1C) and at birth (postnatal day zero: P0; Fig. 1D). In contrast, from P15 to adulthood, robust expression of FXYD2 persisted in 57% of the neurons (Fig. 1E,F). Immunohistochemistry confirmed this observation and revealed that the FXYD2 protein is located in the cytoplasm as well as along nerve fibers exiting the ganglion (Fig. 1G). Altogether these results show that in the DRGs, *FXYD2* expression is induced at postnatal stages around P15, and is restricted to a subpopulation of sensory neurons, suggesting that it might identify specific functional classes.

### *FXYD2* expression is restricted to the TrkB+ mechanoreceptive and Ret+/IB4+ non-peptidergic nociceptive populations

**Figure 2:**
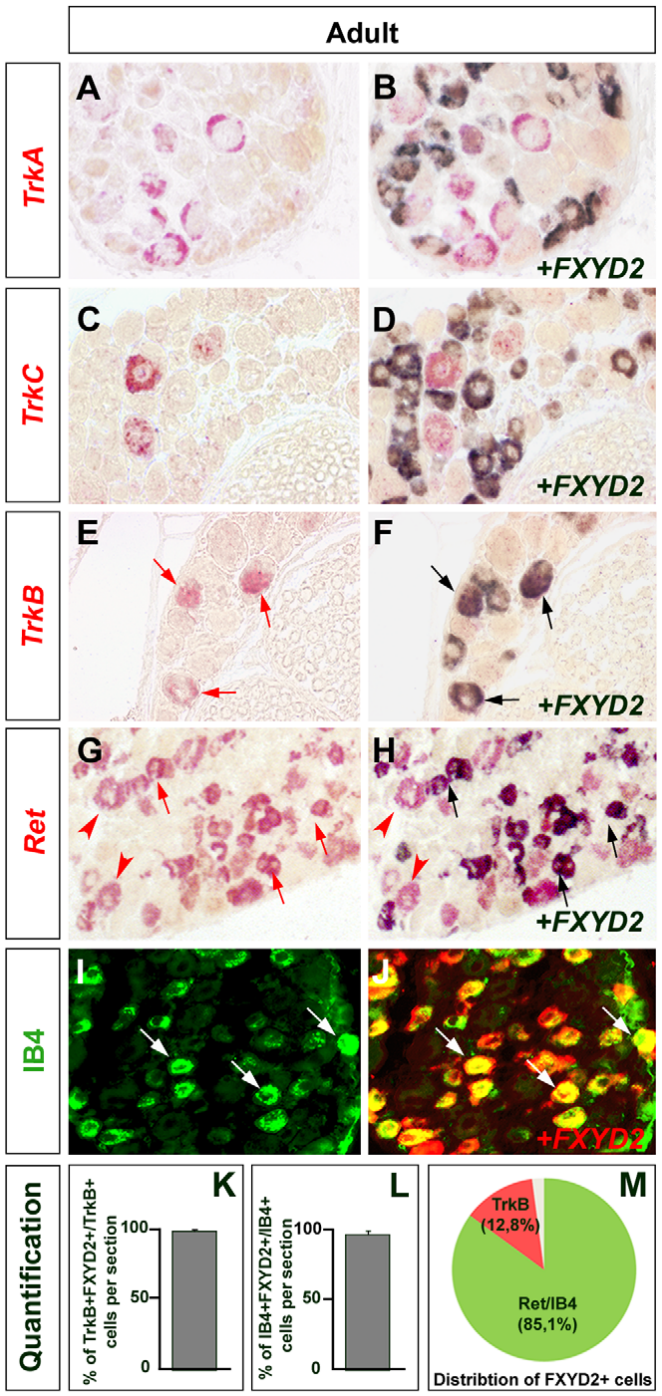
Restricted expression of FXYD2 in TrkB+ mechanoceptive and Ret+/IB4+ non-peptidergic noniceptive neurons within the DRGs. (A–J) Double-labeling for *FXYD2* and *TrkA*, *TrkB*, *TrkC*, *Ret* or IB4 on adult DRG sections. No co-localization is observed between FXYD2 and TrkA or TrkC. Double-positive neurons are detected with TrkB+ mechanoceptors (arrows in E,F) and Ret+/IB4+ non-peptidergic nociceptors (arrows in G–J). Arrowheads in G,H point to large Ret+ mechanoceptive neurons that are *FXYD2*-negative. (K,L) Percentages of TrkB+ (K) or IB4+ (L) neurons expressing FXYD2 showing that virtually all the TrkB+ mechanoceptors and the non-peptidergic nociceptors are FXYD2+. (M) Distribution of FXYD2+ neurons in two main neuronal types: the TrkB+ (representing 13%) and the Ret+/IB4+ (representing 85%) populations.

To test this hypothesis, we analyzed co-expression with *TrkA*, *TrkB*, *TrkC*, *Ret* and the Isolectin-B4 binding molecule (IB4). Double-labeling experiments on adult DRGs sections revealed that virtually no TrkA+ nociceptive (Fig. 2A,B) or TrkC+ proprioceptive/mechanoceptive (Fig. 2C,D) neurons express *FXYD2*. In contrast, we found that *FXYD2* expression is detected in practically all *TrkB*+ mechanoceptive (99%; Fig. 2E,F,K) and Ret+/IB4+ nociceptive populations (97%; Fig. 2G–J,L). In addition, this analysis showed that the FXYD2+ neuronal population mainly segregates into the TrkB+ (representing 13% of the total number of FXYD2+ neurons), and the IB4+ (representing 85%) neurons (Fig. 2M). In keeping with published results, the cell body diameters of *TrkB* expressing neurons were of medium size and those of Ret+/IB4+ neurons were of small size. Ret+ neurons of large cell body diameter consisting in low-threshold mechanoreceptors [6], [7], were mostly negative for FXYD2 (red arrows in Fig. 2G,H). Thus, our expression profile analysis establishes FXYD2 as a new specific marker of TrkB+ mechanoreceptors and non-peptidergic Ret+/IB4+ nociceptors.

### Proper expression of *FXYD2* in non-peptidergic nociceptive neurons requires the transcription factor Runx1 and Ret signalling

**Figure 3:**
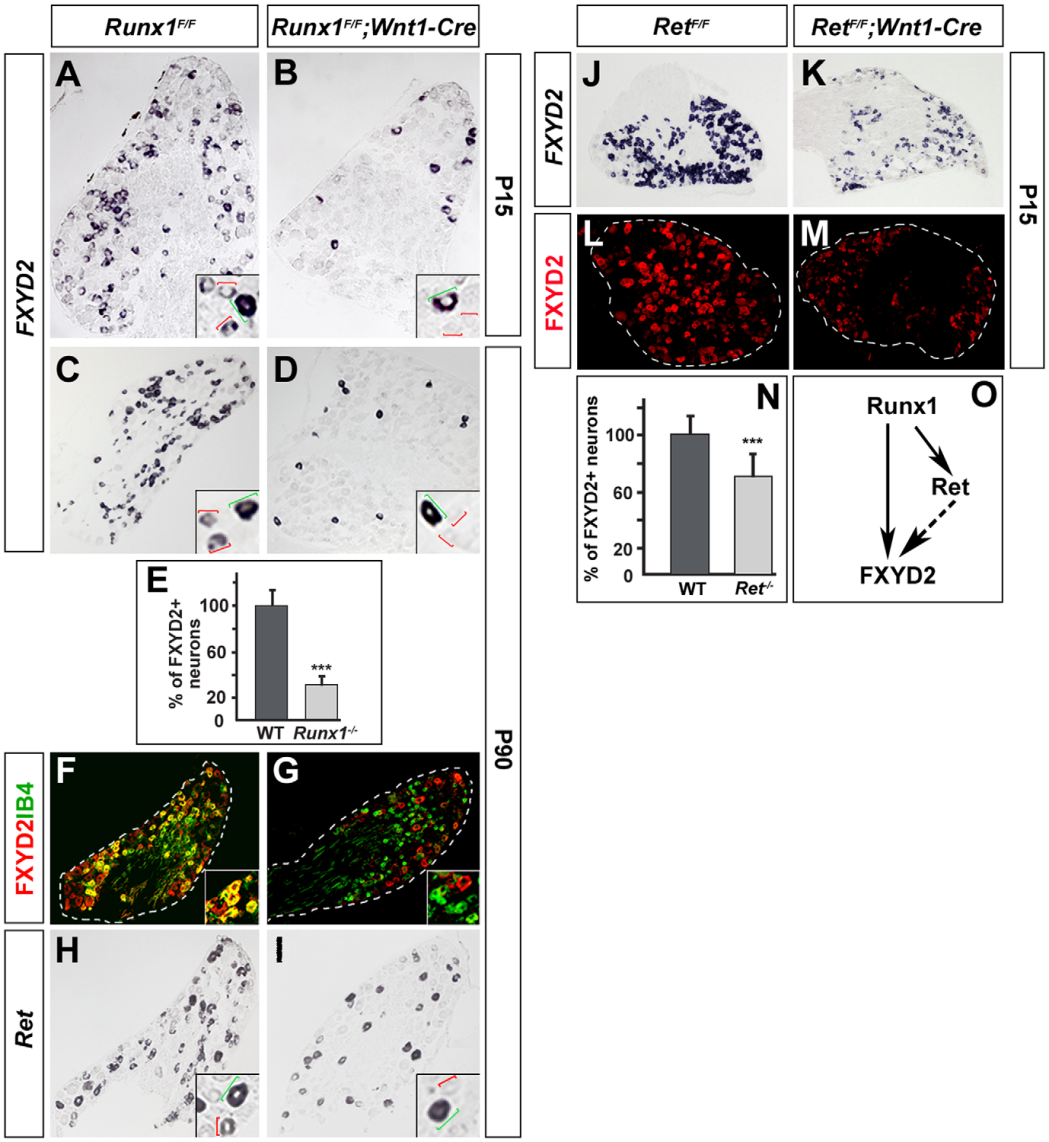
*FXYD2* expression depends on Runx1 and Ret signaling in non-peptidergic nociceptors. (A–D) *FXYD2* in situ hybridization on adult DRG sections from control (*Runx1^F/F^*; A,C) and mutant (*Runx1^F/F^;Wnt1Cre*; B,D) animals at P15 (A,B) and P90 (C,D). Insets show higher magnification. In control (A,C), small and larger (respectively, red and green brackets in insets) diameter neurons are detected, while in *Runx1* mutants at both stages (B,D) only the large diameter population expresses *FXYD2* (green brackets in insets). (E) Quantification of the proportions of FXYD2+ neurons at P90 showing a reduction of 69% in *Runx1* mutants. (F,G) Double-labeling for FXYD2 and IB4 on adult DRG sections from control and Runx1 mutant animals at P90, showing a loss of FXYD2 specifically in the IB4+ population in the mutants. Insets show higher magnifications. (H,I) *Ret* in situ hybridization on DRG sections from control (H) and *Runx1* mutant (I) animals at P90. Insets show higher magnification. *Ret* expression is lost in small diameter nociceptors (red brackets in insets) and persists only in large diameter mechanoceptive neurons (green brackets in insets) in *Runx1* mutants. (J–M) *FXYD2* in situ hybridizations (J,K) and immunochemistry (L,M) on DRG sections at P15 from control (*Ret^F/F^*; J,L) and *Ret* mutants (*Ret^F/F^;Wnt1-Cre*; K,M) showing a reduced number of FXYD2+ neurons and expression intensity in the mutants. (N) Quantification of the relative number of *FXYD2+* neurons showing a reduction of 30% in *Ret* mutants. (O) Epistatic relationships between Runx1, Ret and FXYD2 in non-peptidergic nociceptors. Runx1 controls (directly or indirectly) the onset of FXYD2 expression partly through Ret regulation. Ret signaling seems involved in ensuring proper levels of *FXYD2* and in its maintenance at subsequent stages (dashed arrows; see text).

Recent studies have uncovered the transcription factor Runx1 and Ret signaling as key determinants involved in the differentiation of the non-peptidergic nociceptive neurons [8], [9]. In the absence of *Runx1*, most of the nociceptor population maintains markers of the TrkA+ peptidergic lineage and fail to up-regulate a battery of specific non-peptidergic nociceptive markers, including Ret [8] (see Fig. 3H,I). We thus examined P15 and adult (P90) conditional *Runx1* mutant mice in which the *Runx1* gene was invalidated in the peripheral nervous system. In these mutants at both ages the number of *FXYD2*+ neurons was dramatically reduced by 69%, with only medium sized TrkB+ neurons being labeled (Fig. 3A–E; data not shown). In agreement with Chen et al. (2006) [8], the IB4+ neurons were still present in *Runx1* mutants but fail to express FXYD2 (Fig. 3F,G). This shows that Runx1 in involved in the onset of FXYD2 expression in non-peptidergic nociceptors.

Absence of *Runx1* also leads to down-regulation of *Ret* expression (Fig. 3H,I; [8]). To study putative epistatic relationships between these genes, we examined *Ret* conditional knock-out mice [10] in which *Ret* was eliminated in neural crest derivatives [6]. In these mutants at P15, we found a marked decrease in both the numbers of *FXYD2*+ cells (−30%) and the intensity of expression (Fig. 3J,K,N), a result also visible at the protein level (Fig. 3L,M). Because the mutants die between 2–3 weeks postnatal, we could not study whether at later stages *FXYD2* expression is completely abolished in this genetic background. Nevertheless, this result shows that Ret influences *FXYD2* expression in the non-peptidergic nociceptive neurons. Altogether, our data suggest that Runx1 controls (directly or indirectly) *FXYD2* expression in a Ret-dependent and a Ret-independent manner (Fig. 3O) in the non-peptidergic nociceptors.

### *FXYD2* is down-regulated after sciatic nerve axotomy

**Figure 4:**
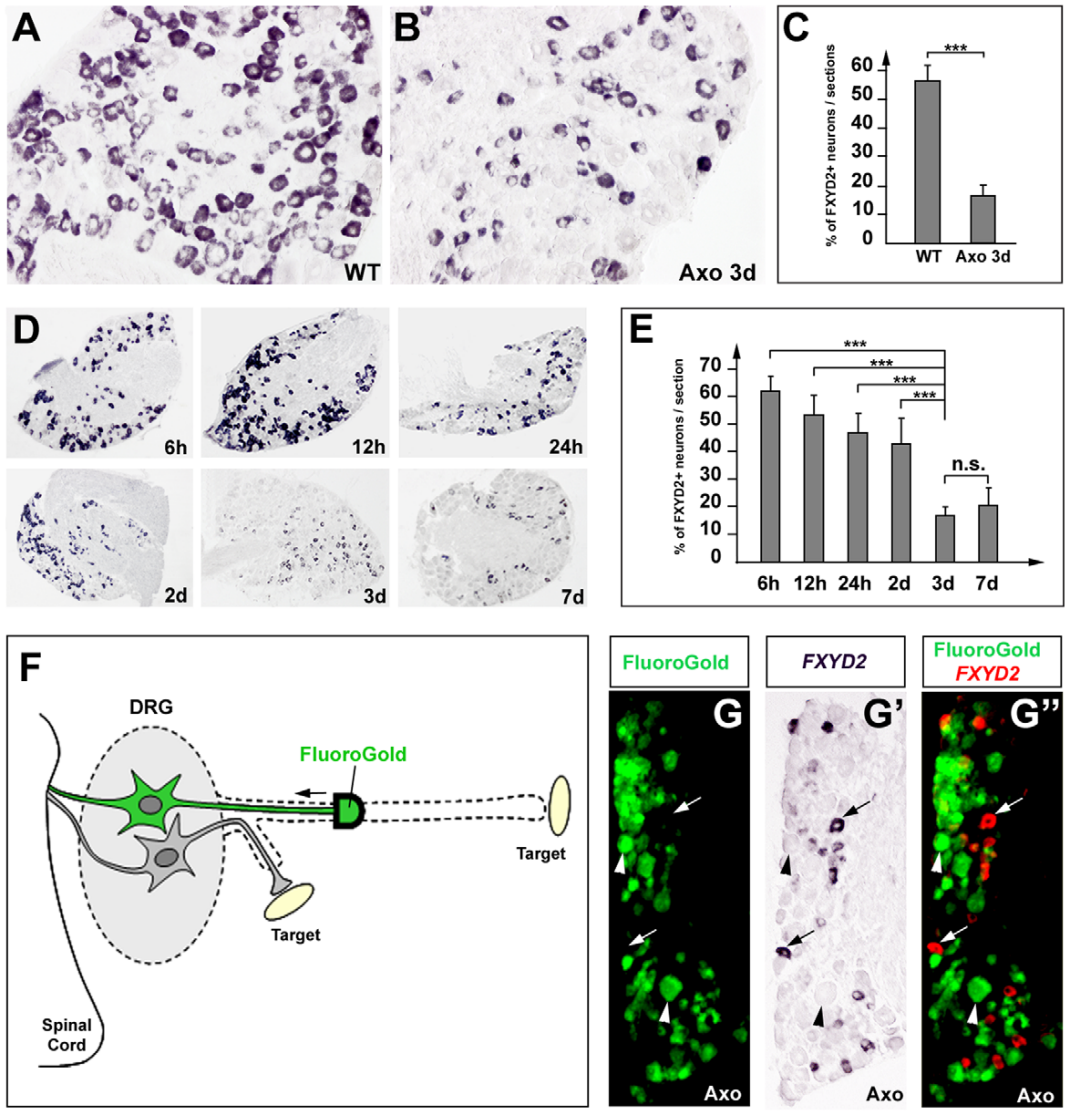
Loss of *FXYD2* expression in L4/L5 DRG neurons after sciatic nerve axotomy. (A, B) *FXYD2* in situ hybridization on naïve DRGs (A) and injured DRGs 3 days post-axotomy (dpa) (B). (C) Quantification of the percentage of *FXYD2*+ neurons in naïve and axotomized DRGs 3 dpa, showing a reduction from 57% to 16% after lesion of the sciatic nerve. (D, E) Time course analysis of *FXYD2* expression in the DRGs from 6 hpa to 7 dpa (D). Quantification reveals a major decrease between 2 and 3 dpa that remains stable at 7 dpa (E). (F) Scheme illustrating retrograde labeling of axotomized DRG neurons with Fluorogold. (G–G″) Combined *FXYD2* in situ hybridization and FluoroGold staining on DRG sections 3 dpa. Virtually no double-positive cells are found. Arrows and arrowheads point to FluoroGold-negative/FXYD2+ and FluoroGold+/FXYD2-negative neurons, respectively.

To gain further insights into the mechanisms regulating *FXYD2*, we took advantage of our SAGE and real-time PCR analysis showing a drastic reduction of *FXYD2* expression after sciatic nerve axotomy (see Fig. 1A). First, we quantified the number of neurons expressing *FXYD2* as a percentage of total neurons in L4/L5 ganglia of normal and axotomized animals. This percentage dropped from 57% in the normal DRG to 16% in the axotomized DRGs, three days post-axotomy (pa) (Fig. 4A–C). Second, time-course analysis carried out at 6 hpa, 12 hpa, 24 hpa, 2 dpa, 3 dpa and 7 dpa (Fig. 4D,E) revealed that *FXYD2*+ neurons began to diminish by 2 dpa (from 61% to 43%). At 3 dpa and 7 dpa this percentage had fallen to 16% and 20%, respectively. The fact that *FXYD2*+ neurons were still detected even after a long period after axotomy suggested that either one population is insensitive to the nerve injury regarding *FXYD2* expression, or that the remaining *FXYD2*+ neurons were not axotomized. To test this, we performed retrograde labeling with Fluorogold from the cut end of the sciatic nerve to unambiguously identify axotomized neurons, combined with *FXYD2* labeling (Fig. 4F). This showed that *FXYD2* expression was absent in virtually all neurons that had taken up Fluorogold (Fig. 4G–G″). Thus, *FXYD2* is down-regulated specifically in axotomized DRG neurons and this down-regulation occurs in both the TrkB+ and IB4+ sub-populations.

### Ret receptor ligands prevent the axotomy-induced down-regulation of *FXYD2* in vitro and in vivo

**Figure 5:**
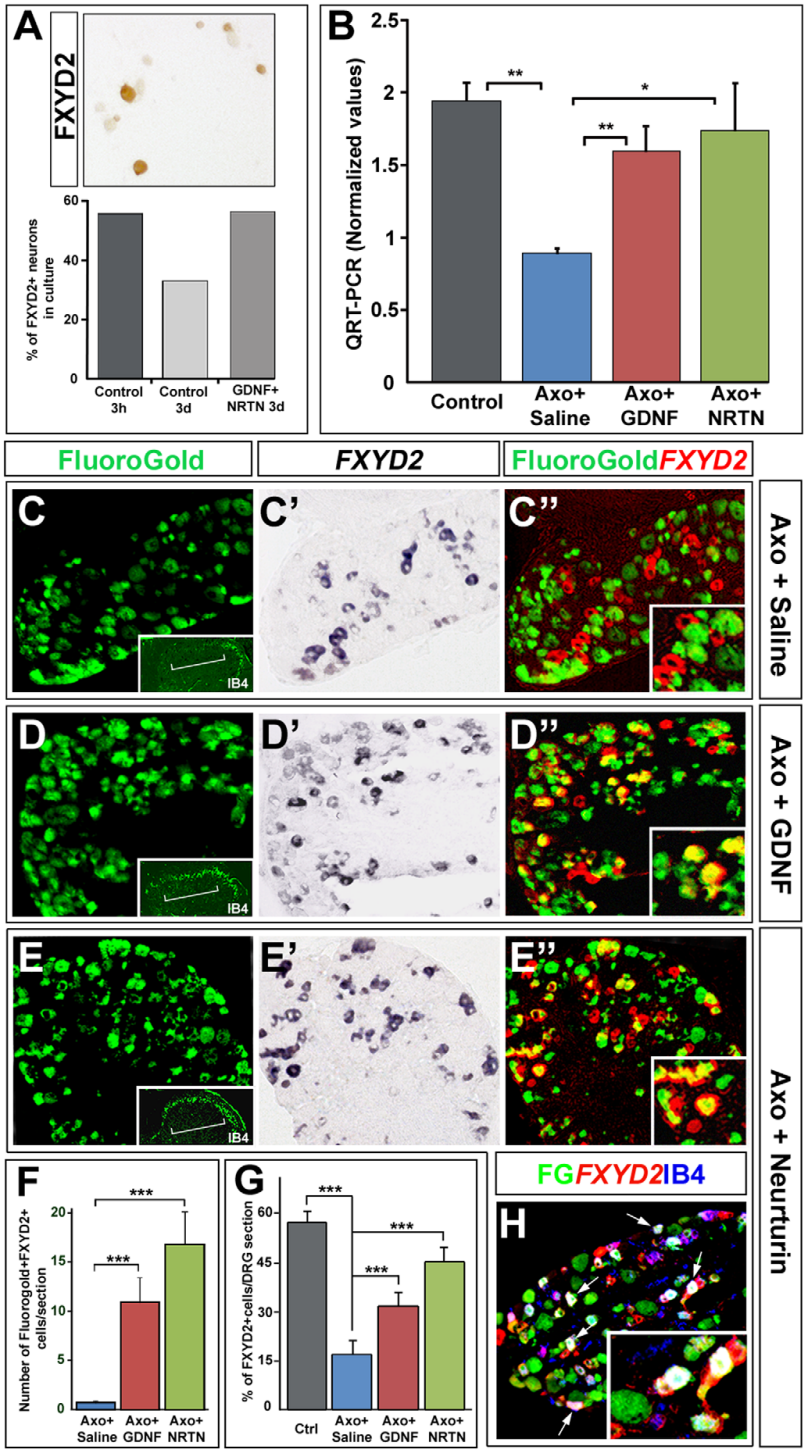
GDNF family ligands influence *FXYD2* expression in adult DRG neurons in vitro and in vivo. (A) Quantitative analysis of FXYD2-expressing neurons in DRG cultures in the presence or absence of GDNF/NRTN. The picture is representative of a neuronal culture stained with the anti-FXYD2 antibody revealed with DAB as a substrate. On the graph is reported the proportion of FXYD2+ neurons after 3 h in culture, 3 days in culture without added factors, or 3 days in culture with GDNF/NRTN (10 ng/ml each). *FXYD2* expression was efficiently maintained by addition of factors. (B) QRT-PCR for FXYD2 on L4/5 DRGs dissected from control animals or mice axotomized and injected intrathecally either with saline, GDNF or NRTN solutions. (C–E″) Combined *FXYD2* in situ hybridization and FluoroGold staining on adult DRG sections from mice axotomized and injected either with saline (C–C″), GDNF (D–D″) or NRTN (E–E″) solutions during 3 days. Double-labeled neurons are virtually absent with saline injection, while they are numerous after GDNF and NRTN treatments. Insets in C″, D″ and E″ show higher magnifications. Insets in C, D and E represent injection quality controls showing IB4 staining on hemisections of the dorsal spinal cord (brackets) ipsilateral to the axotomy, that is normally lost after axotomy and saline injection, but rescued with GDNF or NRTN [11], [12]. (F) Quantification of FluoroGold+/FXYD2+ neurons in the indicated conditions, showing that GDNF family ligands efficiently maintain *FXYD2* in injured neurons. (G) Quantification of the proportion of FXYD2+ neurons per DRG section in naïve animals (Ctrl) or in axotomized mice injected either with saline, GDNF or NRTN solutions. *FXYD2* is normally expressed in 57% of the DRG neurons and in 16% after axotomy and saline injection. In GDNF and NRTN injected mice, this proportion reaches 32% and 44%, respectively. (H) Triple-labeling for FXYD2, IB4 and FluoroGold (FG) on adult DRG sections from axotomized mice treated with NRTN. Presence of triple-labeled cells (white arrows) shows that FluoroGold+/FXYD2+ neurons are IB4+ nociceptors. Inset show higher magnification.

The down-regulation of *FXYD2* in axotomized sensory neurons suggested that peripheral signals might allow the maintenance of its expression. According to our expression pattern and mutant mice analyses, GDNF family ligands appeared as good candidates notably for the IB4+/Ret+ nociceptive population. To test this, we first cultured adult DRG neurons in the presence or absence of GDNF and NRTN and evaluated the percentage of FXYD2+ neurons. GDNF and NRTN administered for 3 days effectively maintained *FXYD2* expression at the levels observed at one day in culture (56% of neurons), whereas in the absence of GDNF family ligands the levels had dropped to 33% (Fig. 5A). This prompted us to investigate whether GDNF family ligands could also maintain *FXYD2* expression *in vivo* by carrying out unilateral axotomy of the sciatic nerve of adult mice and intrathecal application of GDNF, NRTN or saline solutions in the subarachnoidal space. Efficiency of the injections on each animal was systematically monitored by analyzing IB4 staining in the dorsal horn of the spinal cord, which is normally lost after axotomy, but restored after injections of GDNF family ligands (insets in Fig. 5C,D,E and data not shown; [11], [12]). In addition, the retrograde tracer Fluorogold was administered to the cut stump to identify axotomized neurons. First, we showed by QRT-PCR that subarachnoidal injections of GDNF or NRTN reversed the decrease in *FXYD2* transcripts observed after axotomy combined with saline injection (Fig. 5B). To follow these changes at the cellular level, we carried out co-labeling analysis for *FXYD2* and Fluorogold on sections of L4/L5 DRGs treated under the various conditions described above (Fig. 5C–G). As expected, in DRGs from mice injected intrathecally with saline solution alone, virtually no Fluorogold+ (i.e. axotomized) neurons contained *FXYD2* mRNA (Fig. 5C–C″,F). In contrast, application of GDNF (Fig. 5D–D″,F) or NTRN (Fig. 5E–E″,F) resulted in the appearance of numerous double Fluorogold+/*FXYD2*+ neurons (Fig. 5D–G). Moreover, by triple labeling for FXYD2, IB4 and Fluorogold, we showed that Ret ligands maintain the expression of *FXYD2* in the IB4-lectin binding neuronal population (Fig. 5H; data not shown). It is of note that in our experimental paradigm, rescue of FXYD2 expression in axotomized neurons with NRTN seemed slightly more efficient than with GDNF, the percentage of *FXYD2*+ neurons per DRG section increasing from 16% with saline solution to 32% with GDNF and 44% with NRTN (Fig. 5G). Altogether, our results show that in vivo administration of neurotrophic factors of the GDNF family prevents the axotomy-induced down-regulation of *FXYD2* in IB4+-nociceptors and support the view that Ret signaling is necessary for the maintenance of *FXYD2* in adult non-peptidergic nociceptors.

## Discussion

Here, we uncover the Na,K-ATPase modulator FXYD2 as a novel specific marker restricted exclusively to the TrkB+ mechanoreceptors and the IB4-lectin binding non-peptidergic nociceptors of primary somato-sensory neurons. Strikingly, FXYD2 is not ubiquitous in the DRGs, suggesting that the composition of the Na,K-ATPase complex diverges from a sensory neuronal type to another and that the expression of the FXYD family members is finely regulated. Our analysis reveals some aspects of the regulatory mechanism leading to the restricted expression of *FXYD2* in the DRGs, notably in the non-peptidergic nociceptive neurons. Indeed, in this population our data point to roles for Runx1 and Ret in the initiation of *FXYD2* expression at early postnatal stages, and its maintenance in the adult. A role in initiation is demonstrated by the fact that, in P15 and adult DRGs from *Runx1* mutant mice, *FXYD2* expression is completely lost in the IB4+ population. In *Ret* mutants at P15, however, the numbers of *FXYD2+* neurons and the intensity of expression are diminished, indicating a partial dependence on Ret signaling at this stage. Because of the relative early lethality of the *Ret* mutation used in this study, we could not analyze whether *FXYD2* expression is completely abolished at later stages in these mutants. Nevertheless, the importance of Ret signaling for the maintenance of *FXYD2* expression is supported by our results on axotomized neurons. Indeed, administration of exogenous GDNF family ligands of the Ret receptor to injured DRG efficiently restores its expression in axotomized IB4-binding nociceptive neurons. Thus, our results show that *FXYD2* belongs to a group of genes that depend on both Runx1 and Ret signaling for their correct expression [9].

Our rescue experiments on axotomized neurons also further illustrate the influence of periphery-derived cues in maintaining the integrity of DRG neurons. Retrograde signaling by neurotrophins controls many aspects of sensory neuron development and adult function [13]. Similar to neurotrophins, in adult rodents, peripherally injected I^125^ labeled GDNF family ligands are internalized and retrogradely transported to the DRG and accumulate in neurons that express the appropriate receptors [14], and several studies have shown that exogenously-supplied GDNF both normalizes gene expression in injured DRG neurons and exerts analgesic effects in models of neuropathic pain in rodents [15], [16]. In this context, the restoration of *FXYD2* expression by GDNF ligands could have therapeutic relevance.

The molecular mechanisms involved in the control of *FXYD2* expression in the TrkB+ population remains to be determined. The roles of TrkB ligands such as BDNF or NT-4 in the post-traumatic processes in sensory neurons are much less well-defined than those of GDNF ligands. It has been shown, for example, that BDNF production actually increases in Schwann cells at the injury site or in DRG neurons themselves, in several models of peripheral nerve injury (reviewed in [17]).

The finely regulated expression pattern of FXYD2 in normal mouse DRGs and its down-regulation after axotomy raise the issue of its function in sensory neurons. The 7 members of the FXYD family encode gamma-subunits of the Na,K-ATPase which share a common function as modulators of the properties of the pump in various tissues [2], [3]. In neurons, the Na,K-ATPase is essential for maintaining the membrane resting potential by restoration of Na+ and K+ gradients during the propagation of action potentials. Moreover, lesioned sensory neurons become hyperexcitable and this ectopic activity, which might in part reflect changes in Na,K-ATPase subunits expression and/or regulation, may contribute to neuropathic pain [18]. In line with this, binding of the secreted molecule FSLT1 to the Na,K-ATPase alpha 1-subunit induces a hyperpolarization of the membrane which in turn inhibits synaptic transmission in sensory neurons [19]. FSTL1 is down-regulated after axotomy which leads to neuropathic pain hypersensitivity [20]. So far, the major site of *FXYD2* expression is the kidney where it modulates the affinity of the Na,K-ATPase for Na+, K+ and ATP [21]. In the DRGs however, very little information on FXYD proteins is available. Only FXYD7 has been shown to be actually up-regulated in rat DRG neural cells after axotomy [22]. Our results revealing the down-regulation of *FXYD2* as part of the molecular response to nerve injury support the view that it could be one key regulator involved in the development of neuropathic conditions affecting the somatosensory nervous system.

## Materials and Methods

### Animals and surgery

Procedures involving animals and their care were conducted according to the French Ministry of Agriculture and the European Community Council Directive no. 86/609/EEC, OJL 358, 18 December 1986. The protocols were validated by the Direction Départementale des Services Vétérinaires de l'Hérault (Certificate of Animal Experimentation no. 34-376,17 february 2009). Runx1^F/F^;Wnt1-Cre mutant animals were obtained as described in [8]. (2006). Ret^F/F^ animals (provided by Dr. C. Baudet; [10]), were crossed with the Wnt1-Cre mouse line [23] as described in [6].

Axotomy was performed on adult C57BL/6 wild type mice deeply anaesthetized by intraperitoneal injection of equithesin [0.6% pentobarbital sodium and chloral hydrate (0.4 ml/100 gbody wt)]. The left sciatic nerve was exposed at the mid-thighlevel and sectioned, and a 3- to 5-mm fragment of the nerve was removed. For back labelling of axotomised DRG neurons, the proximal portion of the sectioned nerve was immersed several minutes in 0,9% saline solution containing 4% of Fluorogold (Fluoro-Chrome Inc., Denver, CO, USA; Fig. 4F). Mice were kept alive for the indicated times before sacrifice and dissection of the DRGs. Saline solution or Neurotrophic factors (GDNF or NRTN) were administered directly into the spinal subarachnoidal space at the S1 level of adult mice using a 30-gauge needle (BD Micro-fine). 700 ng of GDNF or NRTN (AbCys) were injected once a day during 5 days. Animals were then killed and lumbar DRGs were collected and processed for immunochemistry or in situ hybridization.

### Gene profiling using SAGE

SAGE libraries were made on mouse lumbar DRGs (approximately 80–100,000 cells) using the I-SAGE™ Kit (Invitrogen, France) according to the manufacturer's instructions and as described previously [1]. Analysis of the generated data, and in particular, sequence data analysis assessing the quality of the library, extraction of tag sequences from concatemers, their annotations and analysis of their distributions was carried out using bioinformatic tools developed by Skuld-tech (http://www.skuldtech.com) as previously described [1].

### Real-time PCR

Quantitative RT-PCR was conducted as previously described [1], by using SYBR Green I dye detection on the Light Cycler system (Roche Molecular Biochemicals). The identity of amplified products was confirmed by sequencing (Genome express, France). The relative amounts of specifically amplified cDNAs were calculated using the delta-CT method [24], [25] on three independent experimental replicates, after normalisation by two stable control genes (polymerase (RNA) II (DNA directed) polypeptide J (polr2j) and DEAD box polypeptide 48 (Ddx48)). The Mann Whitney U-test was used for comparison between groups. p value<0,05 were considered statistically significant.

The following primer pairs were used to generate the PCR products:

Polr2j [GenBank:NM_011293]:

s- ACCACACTCTGGGGAACATC; as- CTCGCTGATGAGGTCTGTGA

Ddx48 [GenBank:NM_138669]:

s- GGAGTTAGCGGTGCAGATTC; as- AGCATCTTGATAGCCCGTGT

The FXYD2 primers amplify a region common to the 2 known FXYD2 transcript variants; FXYD2a [Genbank: NM_007503] and FXYD2b [Genbank:NM_052823] [26]:

s- GGACAGAGAATCCCTTCGAG; as- CCGATTTCATTGGCAGTTG

### Western blot

Western blots on DRG or kidney protein extracts were done as described in [27] using the rabbit anti-C-terminal FXYD2 antibody diluted 1/10000 [28], [29].

### Cell Culture

Adult DRG neurons from wild type animals were dissociated and plated at a density of 15000 neurons per well, on 4-well plates (Nunc) with glass coverslips (CML) precoated with D,L-polyornithine (5 µg/ml) and laminin (5 µg/ml), in a defined culture medium consisting of Neurobasal supplemented with 200 mM glutamine (GIBCO), and 2% B27 (GIBCO). GDNF and NRTN (Abcys) were used at a concentration of 10 ng/ml. The primary cultures were maintained at 37°C in a humidified incubator under 5% CO_2_ during 3 days and then processed for immunochemistry (see below).

### In Situ Hybridization, Double In Situ Hybridization and Immunhistochemistry

*TrkA* and *FXYD2* antisense RNA probes were generated from cDNA sequences that were PCR-amplified from reverse-transcribed total RNA isolated from wild-type adult mouse DRG using the following primers:

TrkA s- TGGCAGTTCTCTTTCCCCTA; as- AAAGCTCCACACATCGCTCT

FXYD2s- GGACAGAGAATCCCTTCGAG; as- CCGATTTCATTGGCAGTTG

Amplified fragments were cloned into the pGEM-T easy vector using the TA cloning kit (Promega).

Probes for *TrkB*, *TrkC* and *Ret* were kindly provided by Dr. E. Castren, Dr. F. Lamballe and Dr. V. Pachnis, respectively. Digoxigenin(DIG)- or Fluorescein-labeled RNA probes were synthesized using the DIG- or Fluorescein-labelling kit (Roche), respectively, according to the manufacturer's instructions.

Simple and double in situ hybridization, and in situ hybridization combined with immunohistochemistry were performed as previously described [30].

For simple staining with isolectin B4, cryosections of spinal cord tissue were blocked in 1% BSA, 0.1% Triton in PBS for 1 h, and then incubated with IB4-Biotin (10 mg/ml, Sigma) and FITC-conjugated ExtrAvidin (Sigma, diluted 1/400). For in situ hybridization combined with IB4-labeling, cryosections were first hybridized with the *FXYD2* DIG-labeled probe and processed for IB4 staining as above. Slides were temporarily mounted in 90% glycerol in PBS and fluorescent images of IB4 labeling were taken. Slides were then washed in PBS, blocked in 20% sheep serum, incubated with an alkaline phosphatase (AP)-conjugated sheep anti-DIG antibody (Roche), and then with a solution of NBT/BCIP (Roche, diluted 1/2000), substrate of the AP, to reveal the FXYD2 staining. In situ hybridization signals were photographed under transluminescent light and converted into red pseudo-fluorescent color. Pictures of the same sections were overlaid to reveal co-labeled cells.

Immunofluorescent staining on frozen sections were performed as previously described [31]. Antibodies used were as follows: goat anti-Ret (R&D Systems, diluted 1∶20) and rabbit anti-C-terminal FXYD2 (diluted 1∶1000; see [28]). Alexa Fluor-594-or Alexa Fluor-488-conjugated secondary antibodies were used (Molecular Probes, diluted 1∶1000 and 1∶500, respectively). For Ret immunostaining, an epitope retrieval step was carried out by immersion of the sections for 15 min at 68°C in sodium citrate buffer (10 mM Sodium Citrate, 0.05% Tween 20 [pH 6]).

Immunohistochemistry on adult DRG neuronal cell cultures was conducted as described [27]. Briefly, fixed cell cultures were incubated 1 hr at RT with the rabbit anti-C-terminal FXYD2 antibody (diluted 1/1500). After several washing steps, the antibody was revealed using the Vectastain ABC kit (Vector). Colour reaction was performed using diaminobenzidine (DAB; Sigma) as substrate.

### Cell counting

The number of neurons expressing the various molecular markers was determined by counting cells with neuronal morphology and clearly identifiable nuclei. A minimum of 4 sections from DRGs were counted from at least 3 animals. The average number of positive neurons per section was determined. When indicated on the graphs, the percentage of neurons expressing a given marker either over the total number of DRG neurons, or over the total number of a defined population, was calculated.
